# Recent Advances in Conductive Polymers-Based Electrochemical Sensors for Biomedical and Environmental Applications

**DOI:** 10.3390/polym16111597

**Published:** 2024-06-04

**Authors:** Youheng Pan, Jing Zhang, Xin Guo, Yarou Li, Lanlan Li, Lijia Pan

**Affiliations:** 1 College of Mechanical and Electrical Engineering, Henan Agricultural University, Zhengzhou 450002, China; 2 Collaborative Innovation Center of Advanced Microstructures, School of Electronic Science and Engineering, Nanjing University, Nanjing 210093, China

**Keywords:** conductive polymers, electrochemical sensor, biomedical application, environmental application

## Abstract

Electrochemical sensors play a pivotal role in various fields, such as biomedicine and environmental detection, due to their exceptional sensitivity, selectivity, stability, rapid response time, user-friendly operation, and ease of miniaturization and integration. In addition to the research conducted in the application field, significant focus is placed on the selection and optimization of electrode interface materials for electrochemical sensors. The detection performance of these sensors can be significantly enhanced by modifying the interface of either inorganic metal electrodes or printed electrodes. Among numerous available modification materials, conductive polymers (CPs) possess not only excellent conductivity exhibited by inorganic conductors but also unique three-dimensional structural characteristics inherent to polymers. This distinctive combination allows CPs to increase active sites during the detection process while providing channels for rapid ion transmission and facilitating efficient electron transfer during reaction processes. This review article primarily highlights recent research progress concerning CPs as an ideal choice for modifying electrochemical sensors owing to their remarkable features that make them well-suited for biomedical and environmental applications.

## 1. Introduction

### 1.1. Electrochemical Sensor

Electrochemical sensor is a device that utilizes electrochemical principles for the detection of specific chemical substances, and it can be categorized into various types based on their distinct operational principles and application domains. The pH sensors are utilized for the measurement of solution pH [1,2,3]. Biosensors employ biological materials, such as enzymes or antibodies, as recognition elements to detect metabolites or monitor growth state [4,5,6,7]. Gas sensors are employed for the detection of gas concentration [8,9]. Ion-selective electrodes are utilized for the detection of specific ion concentrations [10]. Compared to colorimetric, fluorescent, and other sensors, electrochemical sensors provide fast reaction, high sensitivity, ease of operation, and miniaturization. Furthermore, real-time detection is possible when paired with microchannel technology. Compared to many electrochemical sensor research studies, the forms of commercialization are significantly lower. The most prevalent commercial electrochemical sensors should be blood glucose meters, which can assist patients in understanding their blood glucose levels more quickly. Furthermore, commercial electrochemical sensors include gas sensors for ammonia, formaldehyde, and oxygen, as well as ion-selective electrodes for pH, nitrate, and potassium plasma in water. Commercial applications are restricted, owing to the drawbacks of electrochemical sensors. Firstly, there is a high reliance on environmental factors. Environmental factors such as humidity, air pressure, and temperature all have an impact on electrochemical sensor performance. For example, increasing humidity causes decreased oxygen permeability and sensor performance. Air pressure fluctuations can potentially have an effect on electrochemical process stability. At high temperatures, the electrochemical sensor’s REDOX reaction rate accelerates, whereas at low temperatures it slows down. Because electrochemical sensors are extremely sensitive to temperature, they are typically temperature adjusted internally to keep the temperature as steady as feasible. Secondly, various factors in complicated environments can interfere with electrochemical sensors, reducing their accuracy and dependability. Furthermore, the electricity of electrochemical sensors is particularly susceptible to pollution and corrosion, which reduces their sensitivity and reaction time. In practical applications, electrodes must be cleaned and maintained on a regular basis to ensure proper operation. All of these facts affect the accuracy and dependability of the electrochemical sensor, limiting its commercial usage. To mitigate these effects while using an electrochemical sensor, the testing environment must be stable. Furthermore, electrochemical sensors must be calibrated while in use to ensure accurate and consistent measurement findings. Calibration is the process of comparing a sensor’s response to a known standard or reference material. The specific calibration techniques vary depending on the sensor type and target analyte. Calibration is normally achieved by creating a reference solution with a known concentration of an analyte and testing the sensor’s response under controlled conditions. Standard calibration materials are often certified reference materials (CRMs) or standard reference materials (SRMs) issued by a certified agency, such as the National Institute of Standards and Technology (NIST). Furthermore, numerous studies aim to enhance the anti-interference capabilities of electrochemical sensors. Selective membranes, for instance, have been employed to lessen chemical influence. To mitigate the effects of the aforementioned factors on sensor performance as much as possible, a variety of nanomaterials with environmental stability and particular recognition properties are used to further modify the electrode.

The typical configuration of an electrochemical sensor comprises a working electrode (WE), a reference electrode (RE), and a counter electrode (CE) [11]. The WE serves as the electrode that undergoes electrochemical reactions with the substance to be detected, while the RE is utilized to provide a stable potential reference, and the CE is employed for current measurement. The research on electrochemical sensors primarily focuses on two key aspects: identifying a suitable recognition element for specific substance detection and enhancing the working electrode interface through nanomaterial modifications. The recognition element plays a crucial role in the specific identification of electrochemical sensors and can effectively improve the anti-interference of the sensor. Furthermore, it is of paramount importance to conduct further research on materials for modifying the WE interface. Typically, the WE is an inorganic metal electrode or a printed electrode, and employing nanomaterials for modification not only expands the planar electrode into three dimensions but also significantly increases its specific surface area. This provides ample sites for high-density loading of recognition elements and greatly enhances the likelihood of electrochemical reactions occurring on the electrode surface. Additionally, due to their excellent conductivity, nanomaterials facilitate rapid recording of reaction signals by providing efficient electron transfer pathways during the reaction process. Commonly utilized modified nanomaterials comprise of nanoparticles, carbon nanotubes, molecularly imprinted polymers, or conductive polymers [12,13,14,15,16,17,18].

### 1.2. Conductive Polymers

**Figure 1:**
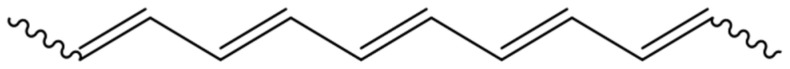
Structure of polyacetylene, showing the main chain containing conjugated double bonds.

Polymers generally refer to compounds composed of polymer chains, which typically exhibit relative molecular weights ranging from several thousand to several million. The electrical conductivity of both natural and synthetic polymers was initially extremely poor, with significant advancements in the synthesis of conductive polymers (CPs) not occurring until the 1970s [19]. The distinguishing feature of CPs lies in the presence of a conjugated main electron system, enabling them to be doped for achieving conductivity levels surpassing 1000 S·cm^−1^. These materials demonstrate conductivity during oxidation (positive doping or p-doping) or reduction (negative doping or n-doping), which can be attributed to the presence of alternating single and multiple bonds (double or triple bonds) as illustrated in Figure 1. The π electrons associated with multiple bonds are delocalized throughout the extended region of the conjugated structure, rendering the polymer susceptible to oxidation or reduction through doping processes. The doping of π-conjugated systems reduces structural and morphological disorder, thereby enhancing conductivity from the interface between insulating materials and semiconductors towards metallic behavior, often resulting in an increased level of conductivity [20,21]. The conductivity of CPs in their neutral state typically ranges from 10^−6^ to 10^−10^ S·cm^−1^, but can reach values up to 10^5^ when doped.

The synthesis of CPs can be achieved through various methods. The most commonly employed synthetic principle is oxidative coupling, which involves the oxidation of monomers to generate cationic free radicals that subsequently undergo coupling reactions to form cations, leading to polymer formation in a repetitive manner. Due to its simplicity and reproducibility, electrochemical synthesis has become the favored and widely accepted approach for fabricating CPs. Another advantageous feature of electrochemical polymerization lies in its ability to operate at room temperature, while allowing precise control over the thickness of thin films through manipulation of potential or current profiles. The electrochemical polymerization of CPs typically employs one of these approaches: (1) constant current or constant current density; (2) constant potential or fixed voltage; (3) potential scanning/cycling or cyclic voltammetry techniques. Commonly used anode materials include chromium, gold, nickel, palladium, and titanium platinum-coated electrodes, as well as indium tin oxide-coated glass plates. Additionally, semiconductor materials such as n-doped silicon [22], cadmium sulfide [23] and semi-metallic graphite [24] are employed for the electrochemical growth of CPs films. This synthetic approach enables the preparation of independent, homogeneous, self-doped thin films while also allowing for copolymerization and grafting reactions to occur if desired.

**Figure 2:**
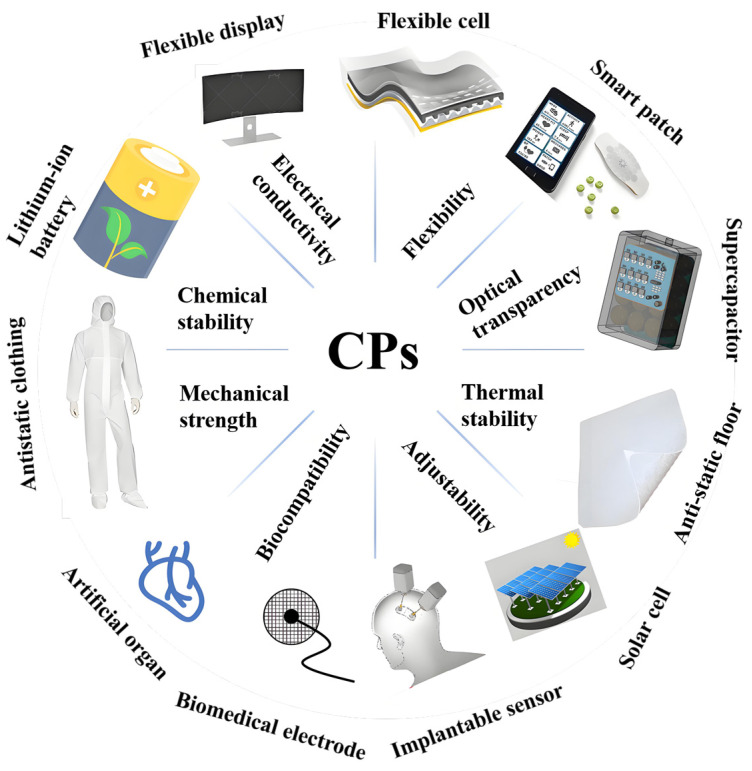
CPs are widely used in various fields because of their many characteristics.

CPs have many excellent properties, such as electrical conductivity, flexibility, chemical stability, optical transparency, mechanical strength, thermal stability, and biocompatibility. These properties make CPs have wide application potential in flexible electronic devices, sensors, optoelectronic devices, biomedical devices, anti-static materials and so on. At the same time, the structure of CPs can achieve specific performance requirements by regulating the chemical composition and structure of the polymer, making it suitable for different application fields (Figure 2).

#### 1.2.1. Polypyrrole(PPy)

**Figure 3:**
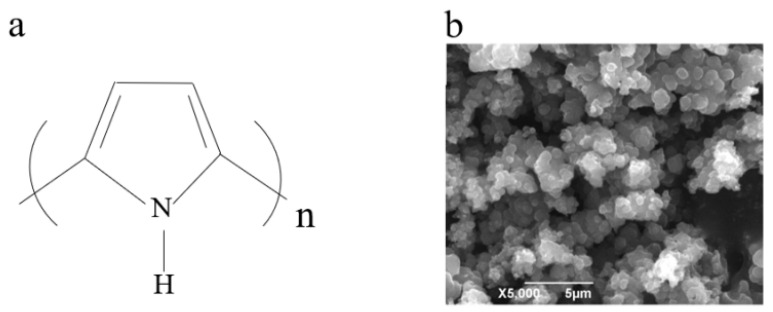
(**a**) Chemical structure diagram of PPy. (**b**) SEM image of PPy. Reprinted with permission from ref. [29].

The basic structure of PPy comprises recurring pyrrole units, which encompass nitrogen atoms and benzene rings in their chemical composition (Figure 3). These pyrrole units are interconnected through conjugated structures, giving rise to polymer chains with conductive properties. PPy stands out among various CPs due to its readily oxidizable monomer, water solubility, commercial availability, lightweight nature, affordability, and biocompatibility [25]. Moreover, PPy demonstrates exceptional conductivity, flexibility, environmental stability, and redox properties. Consequently, it has been widely employed in various biochemical and electrochemical devices [26,27,28]. The combination of PPy with other materials such as carbon-based materials and metal oxides enables the formation of composite materials that exhibit higher electrical conductivity and larger specific surface area. These unique characteristics make PPy an ideal candidate to meet the requirements of electrochemical sensor equipment.

#### 1.2.2. Polyaniline(PANI)

**Figure 4:**
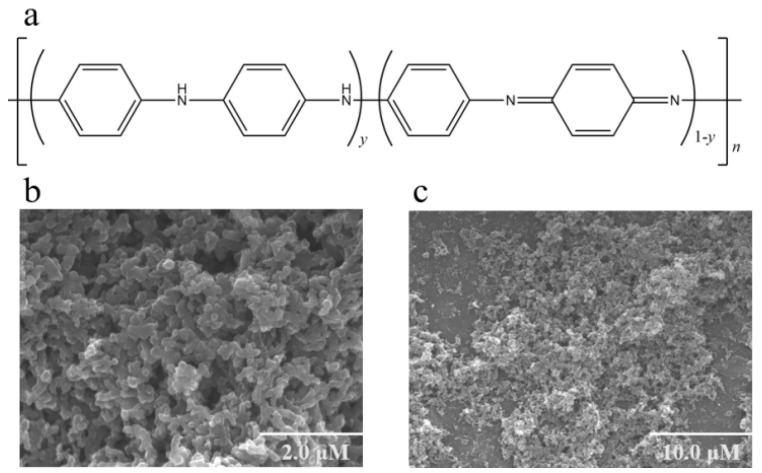
(**a**) Chemical structure diagram of PANI. (**b**,**c**) SEM images of PANI with different scales. Reprinted with permission from ref. [32].

The exceptional environmental stability, high processability, adjustable electrical conductivity and optical properties render PANI as one of the most promising conjugated CPs [30]. The widely accepted structural formula for conductive PANI, proposed by MacDiarmid in 1987, is based on the co-existence model of a “benzene-benzene” chain reduction unit and a “benzene-quinone” alternating oxidation unit, as depicted in Figure 4a. PANI exhibits distinct oxidation states, colors, and electrical conductivity based on the composition of these two structural units, which can interconvert. Since conjugated polymer compounds readily undergo REDOX reactions, PANI can be doped through electrochemical or chemical methods to neutralize the embedded ions in the polymer skeleton and the charge on the polymeric main chain. This enables PANI to rapidly and reversibly transition from an insulating state to a conductive state. When protonic acid is used for doping PANI, the imine nitrogen atom on the molecular chain undergoes protonation reaction under the influence of protonic acid, leading to delocalization and migration of positive charges onto the linear conjugate structure [31]. As a result, PANI exhibits excellent conductivity and unique three-dimensional (3D) crosslinking structure (Figure 4b,c).

#### 1.2.3. Polythiophene(PTh)

**Figure 5:**
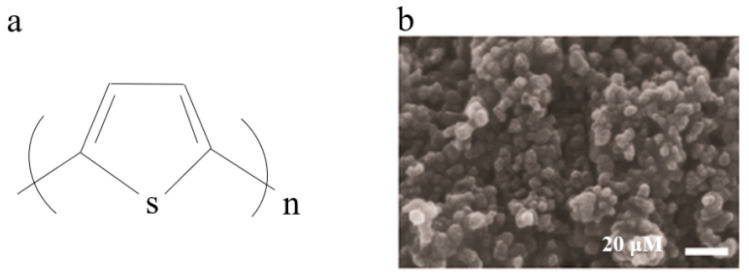
(**a**) Chemical structure diagram of PTh. (**b**) SEM image of PTh. Reprinted with permission from ref. [43].

PTh, along with its undoped state and derivatives, has garnered significant attention in the field of sensor applications due to its discerning barrier characteristics towards specific molecules and high affinity for adsorption [33]. The electronic properties of PTh can be modulated by the introduction of side chain groups or the addition of dopants with band gaps ranging from three electron volts to one [34]. The polymerization and deposition of PTh on large insulating substrates, however, present significant challenges due to their high oxidation potential. Moreover, the oxidized state of doping is highly unstable in air and quickly reverts back to its intrinsic state. Several methods can be employed for preparing polymer films, including solution chemical oxidation, electrochemical oxidation, oxidative chemical vapor deposition, and electrospinning [35,36,37]. The exceptional electrical conductivity, thermal conductivity, and processing stability of P3ATs make them highly regarded as one of the most important types of PTh, such as poly(3-hexylthiophene) (P3HT), poly(3-amylthiophene) (P3PT), and poly(3-butylthiophene) (P3BT) [38,39]. The increase in alkyl chain length within these polymer structures results in a gradual enhancement of phase separation degree, thereby leading to an improved balance between hole and electron transport [40,41,42]. Figure 5 shows the chemical structure diagram and SEM characterization of PTh.

#### 1.2.4. Poly (3, 4-acetylene dioxthiophene) (PEDOT)

**Figure 6:**
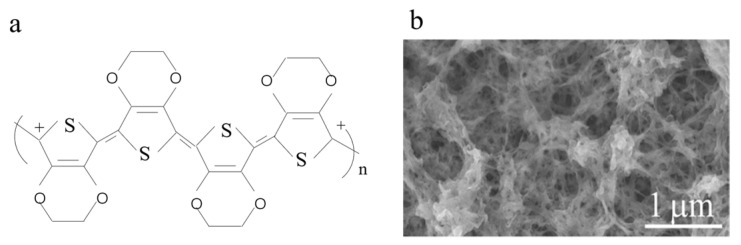
(**a**) Chemical structure diagram of PEDOT. (**b**) SEM image of PEDOT. Reprinted with permission from ref. [51].

PEDOT is a conjugated polymer that achieves charge conduction through a system of conjugated π electrons; it also exhibits excellent electrical conductivity and solubility due to its chemical structure consisting of acetylene dioxthiophene units that form polymer chains through polymerization (Figure 6). Furthermore, it can be compounded with other materials to enhance its properties and adapt to specific application requirements. For instance, hydrophilic surfactants like polystyrene sulfonate (PSS) are often incorporated to improve the water processability of PEDOT film. The combination of PSS and PEDOT results in the formation of a water-soluble PEDOT/PSS mixture [44,45]. Various methods have been employed to improve the conductivity of PEDOT/PSS, such as using polar solvents (e.g., dimethyl sulfoxide, ethylene glycol, and cosolvents [46]) or acids (e.g., chloroplatinic acid, sulfonic acid, and inorganic acid [47,48,49]) to eliminate excessive PSS and induce phase separation or morphological rearrangement. The recent advancement in electrospinning technology has enabled the preparation of flexible conductive PEDOT/PSS nanofibers [50].

#### 1.2.5. Polyacetylene (PA)

PA is a linear conjugated polymer composed of a polyene chain. Being one of the most fundamental organic polymers, the conductivity of PA is significantly influenced by its conformation [52]. Cis-polyacetylene and trans-polyacetylene exhibit conductivities of 10^−9^ S·cm^−1^ and 10^−6^ S·cm^−1^, respectively [53]. However, the conductivity of PA can approach metallic levels (10^4^–10^5^ S·cm^−1^) through p- or n-doping processes [54,55,56]. The practical applications of PA are limited due to its high instability and challenging processing characteristics [57,58]. The methods employed for the preparation of PA encompass catalytic polymerization, non-catalytic polymerization, and precursor-assisted synthesis [59]. Catalytic polymerization techniques are commonly used to produce PA or oligomers, necessitating catalysts with high solubility and selectivity. Radiative polymerization methods, such as glow discharge, ultraviolet or gamma rays, can also be employed to synthesize PA without the need of catalysts or solvents, thereby exhibiting significant potential for future advancements in this field. To improve the conductivity properties, PA is often hybridized or doped with other materials, such as hexamethylene phosphate, quaternary ammonium salt cellulose nanoparticles or gold nanoparticles [60,61,62]. Subsequently, it can be employed in electrochemical biosensors and bioelectrodes.

#### 1.2.6. Poly(p-phenylene vinylene) (PPV)

The chemical structure of PPV consists of a benzene ring and ethylene monomer, where the benzene ring is connected by conjugated double bonds to form a conjugated π-electron system [63]. The molecular structure of PPV contains a large number of π-conjugated bonds, which form a conjugated system that allows electrons to move freely throughout the entire molecule. The conjugated structure enables electrons to efficiently conduct within the polymer chain, resulting in a higher conductivity of the polymer. Moreover, the planar arrangement of PPV facilitates the movement of electrons between molecules, promotes the formation of conjugated structures, and thus improves conductivity. The introduction of additional charge carriers through doping can further enhance the conductivity, while the redox reaction affects its conductivity, which can be adjusted by control [64]. These characteristics make PPV have broad application prospects in fields such as optoelectronic devices and organic light-emitting diodes (OLEDs).

There are numerous varieties of CPs, and we just provide a quick overview of the most widely used ones. In this review, we will look at the biological and environmental applications of these CPs-based electrochemical sensors. Additionally, we analyze the composite methods of CPs with other materials, as well as their structural characteristics, advantages, and exceptional features in sensor technology. This distinctive combination allows CPs to increase active sites during the detection process while providing channels for rapid ion transmission and facilitating efficient electron transfer during reaction processes. The methods for CPs to enhance the active sites during detection include surface chemical modification, nanocomposite, biological fixation, and electrochemical methods. The surface of CPs is chemically modified to introduce active or functional groups, such as carboxyl, amino, hydroxyl, etc. These functional groups can provide active sites, enhance the interaction with the molecules to be measured, and thus improve the sensitivity and selectivity of the sensor. Nanocomposites are formed by combining nanomaterials with conductive polymers. Commonly used nanomaterials include gold nanoparticles, carbon nanotubes, and nano-oxides. These nanomaterials have large specific surface area and special electronic structure, which form additional active sites on the CPs surface and enhance the interaction with the target molecules. Biomolecules (such as enzymes, antibodies, DNA, etc.) are fixed on the CPs surface to construct biosensors. These biomolecules have the ability to specifically recognize target molecules, and when combined with CPs, the biometric recognition performance of the sensor can be enhanced. The active sites were formed on the CPs surface by electrochemical method. For example, electrochemical techniques such as cyclic voltammetry and amperometry can be used to control the REDOX reaction on the surface of CPs to form an active site and enhance its ability to interact with target molecules. These methods can be used individually or in combination to improve the performance of the sensor and achieve highly sensitive and selective detection of the target molecule. Our aim is to showcase the latest advancements in materials science concerning sustainability, scalability, long-term stability, and cost-effectiveness while exploring future directions for polymer synthesis and utilization. These innovative materials hold promise for addressing pressing issues across various fields such as biomedicine and rapid environmental monitoring.

## 2. Biomedical Applications

Electrochemical sensors based on CPs have a wide range of applications in the biomedical field, including biomolecular detection, identification of cancer markers, and drug screening and delivery. These sensors not only enable highly sensitive and selective detection of target molecules in biological samples but also play a crucial role in precise drug release, cell status monitoring, and artificial organ research and development. As such, they significantly contribute to the advancement of biomedical science and clinical medicine.

### 2.1. Biomolecular Detection

#### 2.1.1. pH

The pH level has a significant impact on the human body and plays a crucial role in maintaining the stability and proper functioning of the internal environment. The optimal blood pH ranges from 7.35 to 7.45, and even slight deviations can lead to severe physiological issues [65]. Imbalances in pH, either too alkaline or too acidic, can negatively affect protein function, enzyme activity, metabolism, and cellular processes [66]. The digestive system also relies on specific pH levels for different functions. For instance, stomach acid with a pH of approximately 1–2 aids in food digestion and pathogen elimination [67]. Conversely, the small intestine requires a relatively alkaline environment (around 8) to facilitate enzyme activity and nutrient absorption [68]. The pH of the intracellular and extracellular environments plays a critical role in cell function and metabolic processes. Different cells and tissues have distinct requirements for specific pH ranges, which are essential for maintaining cell membrane stability, regulating enzyme activity, and facilitating ion channel function [69]. In summary, maintaining proper pH levels is crucial for the optimal functioning of the human body’s physiological properties. Consequently, there arises a pressing need to develop a portable device capable of swiftly and accurately detecting pH levels. Electrochemical sensors based on CPs have emerged as the preferred choice owing to their exceptional conductivity, expansive surface area, and selectivity.

**Figure 7:**
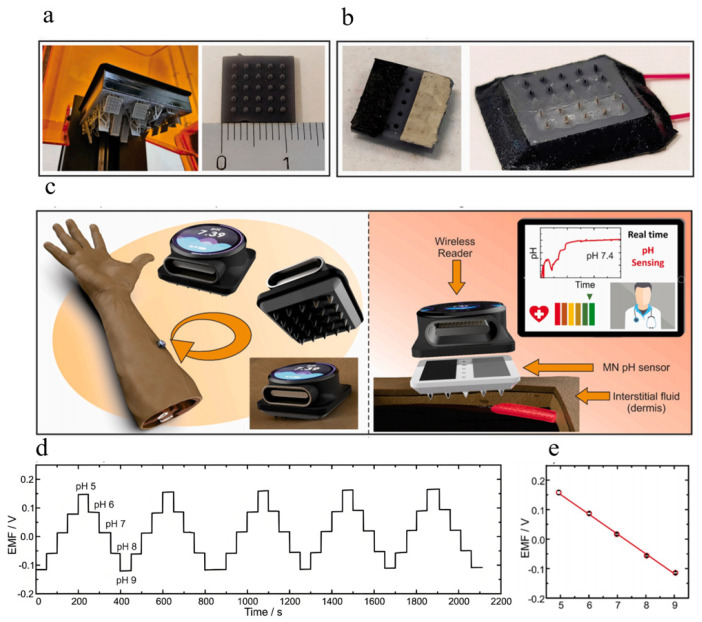
(**a**) Fabrication of the 3D-printed HMNs array. (**b**) The backside and frontside of HMNs-based potentiometric sensor. (**c**) Concept of the MN-based pH sensor pierced on the forearm of a subject for the transdermal monitoring of pH and full connectivity to healthcare service. (**d**) Reversibility test upon increasing and decreasing pH solutions from pH 9 to pH 5 during five cycles. (**e**) Corresponding calibration curves (N = 10). Reprinted with permission from ref. [70].

There are two types of sensors for real-time pH monitoring: in vivo and in vitro. When measuring pH within the body, most researchers opt to measure it through the design and preparation of microneedles that can be inserted into the skin. The development of a manganese-based potential sensor utilizing 3D-printed hollow microneedles (HMNs) is demonstrated by Parrilla (Figure 7a,b) [70]. The 3D-printed microneedle patch was inserted into pig skin using a quasi-membrane model, and its piercing capability was verified. The hollow nanoparticles were subsequently filled with conductive ink to fabricate a set of microelectrodes. Additionally, the WE and RE were suitably modified with PANI and PVA, respectively, in order to enhance the stability of the potentiometer battery. Subsequently, the HMNs sensor was implanted into the subject’s forearm (Figure 7c) for evaluating its capability in monitoring bodily functions. Additionally, comprehensive in vitro characterization was conducted across a wide pH range spanning from pH 5 to pH 9, which aligns with the relevant index range for wound healing [71]. A remarkable performance was achieved, exhibiting a steep recurrence slope of −67.2 ± 1.0 mV·pH^−1^ (N = 10) (Figure 7d,e). The HMNs-based pH sensor demonstrates Nernstian response over an extensive linear range, making it suitable for real-time monitoring of interstitial fluid and wound healing-related pH levels.

**Figure 8:**
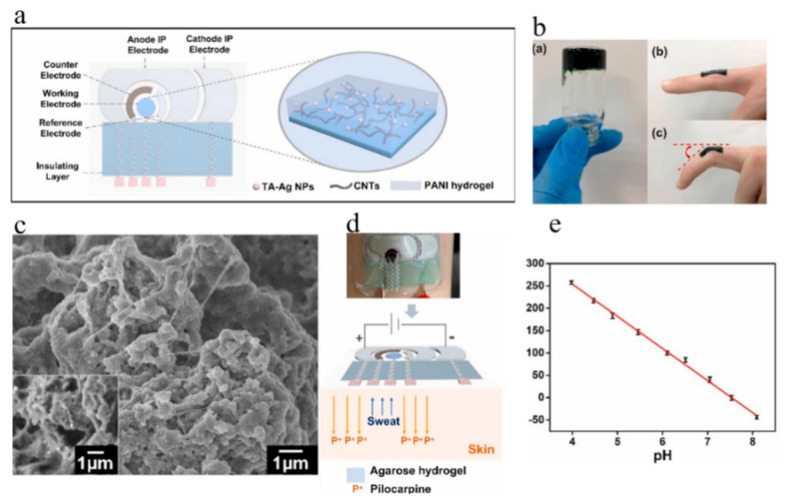
(**a**) Schematic illustration of the wearable sensor. (**b**) Photographs of TA-Ag-CNT-PANI hydrogel (inset a) and the hydrogels attached on the straight or bent fingers (inset b and c). (**c**) SEM of the TA-Ag-CNTPANI hydrogel. (**d**) Schematic diagram of the operation, and the sweat was generated through the ionophoresis delivery of pilocarpine from the anode. (**e**) The linear calibration curve of the OCP response versus pH. Reprinted with permission from ref. [74].

Conversely, numerous researchers monitored pH levels by means of external sweat analysis [72,73,74]. Perspiration can be influenced by environmental conditions, such as temperature and humidity, as well as activity levels and chemical stimuli. While exercise is a reliable method to induce perspiration, meeting the demand for on-demand perspiration analysis in sedentary individuals poses challenges. Therefore, it is necessary to employ appropriate sweat stimulation methods that allow controlled sweat production for analytical applications. Ionophoresis (IP) is a commonly used chemical delivery process involving the application of an imperceptible local electric current to the skin, which stimulates sweat glands to produce sweat (e.g., pilocarpine) [75,76]. The highly integrated and flexible wearable sweat devices developed by Xu enable successful pH detection (Figure 8) [74]. The device is a non-invasive wearable sweat sensing patch comprising an electrochemical sensing system and a pilocarsin-based IP system, which was employed to stimulate sweat secretion. The electrochemical sensor utilized a tannin-silver-carbon nanotube-polyaniline (TA-Ag-CNT-PANI) hydrogel to modify the WE, and the hydrogel film exhibits excellent mechanical elasticity and structural stability. The sensor records the pH response ranging from 3.98 to 8.09, encompassing the entire pH range of sweat, and exhibits a sensitivity of −71.86 mV·pH^−1^.

#### 2.1.2. Glucose

Diabetes is a metabolic disorder with multiple etiologies that manifests as hyperglycemia. The associated risks of diabetes include cardiovascular disease, retinopathy, nephropathy, neuropathy and other complications that can result in blindness, renal failure and serious health consequences [77]. The prevalence of diabetes worldwide has witnessed a rapid surge in recent decades, and it is projected by the International Diabetes Federation that the global number of diabetes cases will escalate to 600 million by 2035 [78]. The presence of elevated blood glucose levels is a prominent characteristic in individuals diagnosed with diabetes, and when the blood glucose levels exceed 6.5 mM, it indicates the likelihood of diabetes [79,80]. Therefore, accurate and timely glucose detection is of paramount importance in safeguarding human lives. Electrochemical techniques have garnered significant attention for their cost-effectiveness, direct quantification capabilities, ease of miniaturization, and suitability for wearable devices compared to alternative methods such as colorimetry, optical coherence tomography, and near-infrared reflectance spectroscopy [81,82,83,84,85].

**Figure 9:**
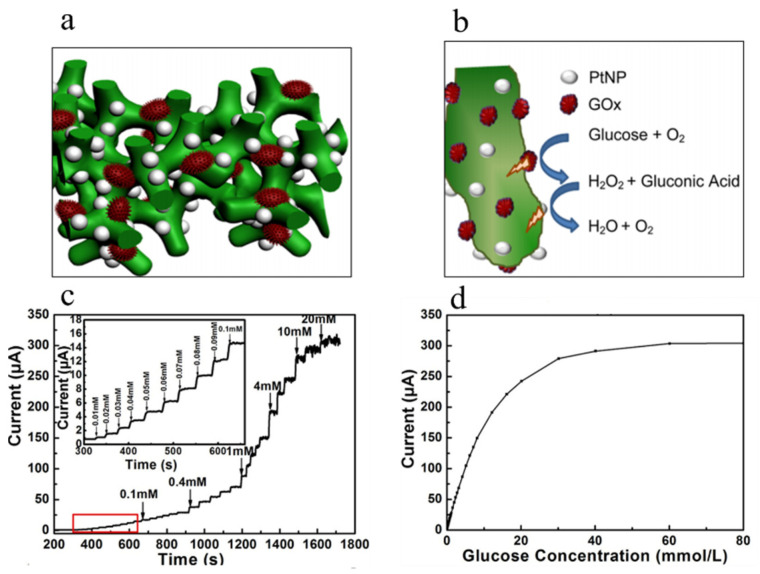
(**a**) The 3D heterostructural diagram depicts the integration of PtNPs/PANI hydrogels, wherein PANI hydrogels function as substrates for immobilizing GOx enzymes and facilitating uniform loading of PtNPs. (**b**) The reaction mechanism of glucose sensor based on PtNPs/PANI hydrogel electrode. (**c**) Amperometric response of the PtNPs/PANI hydrogel electrode after successive addition of glucose in 0.1 M PBS (pH = 5.6) at an applied potential of 0.56 V. Inset: the magnified part of the curve marked with red square. (**d**) The calibration curve for glucose concentrations from 0.001 to 80 mM. Reprinted with permission from ref. [86].

PANI is a promising material for modifying electrochemical sensor electrodes. Zhai developed a highly sensitive glucose sensor using PANI hydrogels [86]. The heterostructure and reaction mechanism of the glucose sensor, which is based on PtNPs/PANI, are depicted in Figure 9, showcasing the synergistic benefits derived from the integration of conductive polymer hydrogels (CPHs) and nanoparticles. The porous cross-linked structure of PANI facilitates the efficient immobilization of glucose oxidases and PtNPs, thereby enhancing the permeability for water-soluble molecules and effectively catalyzing glucose oxidation (Figure 9b). As shown in Figure 9c, the typical current–time response of the electrode at 0.56 V with continuous glucose addition. As the glucose concentration increases, the current increases immediately and quickly reaches a steady state. The average response time of the sensor is as short as 3 s (95% of steady-state current). Figure 9d shows the relationship between current and glucose concentration. In the concentration range of 0.01–8 mM, the steady-state current of the enzyme sensor was linearly correlated with the amount of glucose added to the buffer solution, and the correlation coefficient was 0.993. The calibrated linear portion calculated a sensitivity of up to 96.1 μA·mM^−1^·cm^−2^, higher than previously reported values for sensor electrodes based on platinum and polyaniline composites, polypyrrole, or multi-walled carbon nanotubes (MWCNT).

**Figure 10:**
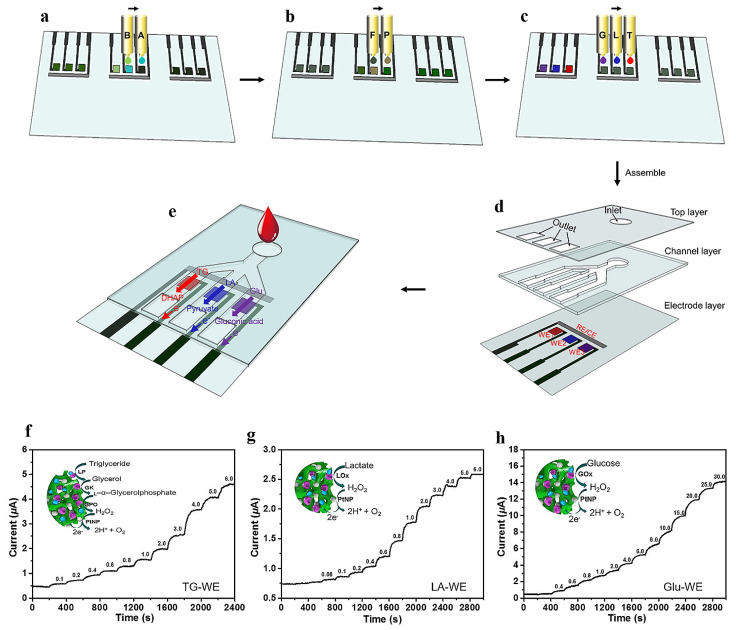
(**a**–**e**) Schematic diagram of design and manufacture of inkjet printing multi-channel biosensor based on conductive PANI hydrogels. (**f**–**h**) Instant current–time response curves of printed biosensors when metabolite solutions with different concentrations were pumped into the channel in an alternating manner. Reprinted with permission from ref. [87].

The integration of miniature sensors on a chip has led to the emergence of multiplexing as a prominent trend in biosensors, enabling simultaneous detection of multiple analytes. However, the precise deposition of electrode materials and selective enzymes on different microelectrode arrays remains a significant challenge for the mass production of multiplexed sensors. Li et al., introduced a three-indicator biosensor based on conductive PANI hydrogels, which was fabricated using an inkjet printing process [87]. The conductive PANI hydrogels were prepared using an all-solution method, enabling their deposition as the interface material through inkjet printing (Figure 10a–e). Subsequently, PtNPs and enzymes were precisely printed onto the designated working electrode. The PANI-based printed biosensor exhibits the capacity to simultaneously detect glucose, lactic acid, and triglycerides. Figure 10f–h records the detection mechanism of triglyceride, lactic acid, and glucose, as well as the instantaneous current–time relationship of the biosensor. It was found that the detection ranges of triglyceride, lactic acid and glucose were 0.1–6 mM, 0.08–5 mM, and 1–25 mM, respectively, and the detection limits were 0.07, 0.06, and 0.2 mM (S/N = 3). The linear range of these sensors can meet the requirements of human metabolic level sensing.

Pan et al., also developed CPs for glucose detection, utilizing a novel composite CPs electrode composed of MXene and conductive PEDOT/PSS hydrogel. They successfully fabricated a non-invasive flexible electrochemical glucose sensor for continuous monitoring of human glucose levels through a simplified one-step synthesis method [88]. The PEDOT–PSS/MXene solution is mixed with ethylene glycol to enhance the polymer chain elongation and facilitate the formation of a homogeneous hydrogel solution. This approach effectively enhances the film-forming properties, flexibility, and stability of the material while mitigating issues related to powder accumulation and spalling. Moreover, this glucose biosensor demonstrated exceptional electrochemical performance in sweat, which exhibited a strong correlation with blood glucose concentration. Therefore, it is anticipated to further enhance the potential of non-invasive blood glucose monitoring.

The enzyme-free glucose electrochemical sensor is one of the sensors utilized for glucose detection. An enzyme-free glucose sensing microneedle was developed by combining PEDOT/PSS with Ag-Pt nanoparticles [89]. The low-cost hydrogel microneedle-continuous glucose meter method employed an expandable dopamine-hyaluronic acid hydrogel for the determination of glucose in dermal interstitial fluid. Platinum and silver nanoparticles were synthesized within a 3D porous hydrogel scaffold to enable non-enzymatic electrochemical sensing of glucose, while the incorporation of a highly aqueous dispersible conductive polymer enhanced the electrical properties of the hydrogel microneedle array, making it suitable for use as a working electrode in the sensor.

#### 2.1.3. Cholesterol

Cholesterol is a vital structural component of both the plasma membrane and nerve cells [90]. The normal concentration of cholesterol in the blood can vary between 5.2 and 6.2 mM, depending on factors such as body weight, age, and sex. Hypercholesterolemia occurs when levels exceed 6.21 mM, which significantly increases the risk of myocardial infarction, atherosclerosis, high blood pressure, and coronary heart disease. Conversely, low cholesterol levels can lead to anemia and liver disease [91]. Therefore, accurately determining cholesterol is crucial for analyzing clinical diseases caused by its abnormality [92,93,94].

The conventional approach to cholesterol detection involves the utilization of an electrochemical cholesterol biosensor, which relies on the activity of cholesterol oxidase (ChOx). The selectivity of this sensor can be significantly enhanced through the incorporation of an enzyme electrode. Alagappan prepared an cholesterol biosensor based on AuNPs-f-MWCNTs-PPy-ChOx/GCE [95]. The biosensor was fabricated through a two-step process, involving the preparation of AuNPs-f-MWCNT via wet chemical method followed by electrical polymerization of pyrrole. PPy serves as a supportive matrix for immobilizing ChOx, while the presence of Au-f-MWCNT enhances conductivity. The PPy-based biosensor exhibited a linear response within the concentration range of 2–8 mM, demonstrating sensitivity and detection limits of 10.12 μA·mM^−1^·cm^−2^ and 0.1 mM, respectively. Li et al., developed and fabricated a biosensor platform based on CPHs [96]. The biosensor can be prepared using an all-solution process, utilizing phytic acid as both crosslinkers and dopants. The PANI hydrogel film can be formed within 3 min, while cholesterol esterase/cholesterol oxidase (ChEt/ChOx) was cross-linked in the PANI hydrogel matrix with the bifunction compound glutaraldehyde, enabling high-density and uniform deposition of the enzyme onto the three-dimensional nanostructure of polyaniline. Due to the unique characteristics of CPHs, such as their high permeability to biological substrates and rapid electron transfer, this biosensor exhibits excellent sensing performance with a wide linear range (0.3–9 mM), high sensitivity, low detection limit, and fast response time (approximately 3 s). Due to the easily scalable workability of CPs, the proposed CPs-based biosensor platform exhibits significant potential as a cost-effective sensor suite for medical monitoring, clinical diagnostics, and biomedical devices. A cholesterol biosensor without the need for enzymes can also be prepared by coating a screen-printed electrode with taurine (TA) modified PEDOT [97]. The sulfonic acid in TA forms electrostatic interactions with the polymerization main chain, exhibiting exceptional stability, high dispersion, and significant surface area adsorption of cholesterol through the induction matrix, thereby enhancing its electrochemical performance. The results demonstrate that the sensor exhibits an extremely low detection limit of 0.95 µM (S/N = 3), enabling rapid detection of low concentrations of cholesterol in various bodily fluids.

#### 2.1.4. DNA and RNA

Although society is making continuous progress, it also faces numerous challenges stemming from these advancements, such as the prevalence of life-threatening diseases that include cancer, genetic disorders, and infectious ailments. The identification and diagnosis of these sicknesses hold paramount importance, thus necessitating the utilization of electrochemical biosensors as potent bioanalytical tools, particularly for nucleic acid detection. Electrochemical DNA (E-DNA) sensors are favored by researchers for their fast response time, accurate measurement and stability, and seamless integration with electronic devices [98,99,100,101,102].

**Figure 11:**
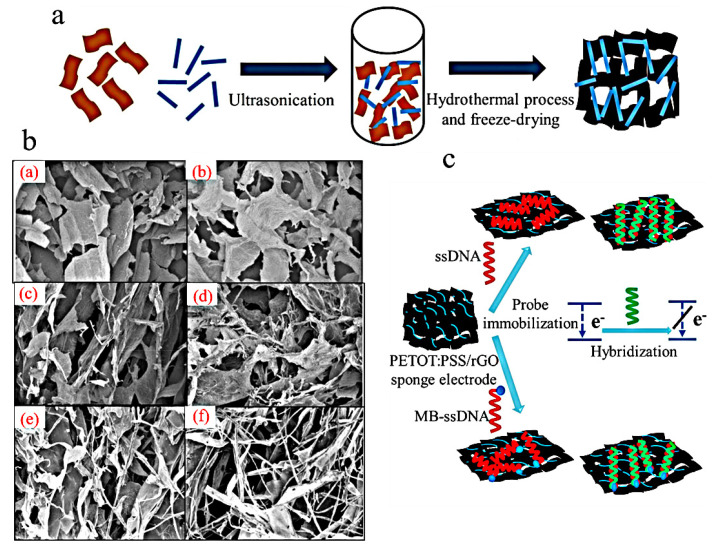
(**a**) Schematic illustration of the preparation of PEDOT–PSS/RGO composite sponge material. (**b**) SEM images of RGO (a), PEDOT–PSS-2/RGO (b), PEDOT–PSS-4/RGO (c), PEDOT–PSS-6/RGO (d), PEDOT–PSS-8/RGO (e), and PEDOT–PSS-10/RGO (f). (**c**) Schematic representation of the immobilization and hybridization of ssDNA and MB-ssDNA probes onto the PEDOT–PSS/RGO composite sponge material. Reprinted with permission from ref. [104].

The application of the E-DNA sensor relies on the process of DNA hybridization [103]. Kiransan et al., developed polymer-supported graphene-based materials utilizing DNA hybridization for electrochemical determination [104]. The first step involved transforming a polymer dispersion of graphene oxide (GO) and PEDOT/PSS with varying compositions into a hydrogel through hydrothermal treatment. Subsequently, a 3D flexible composite sponge material consisting of PEDOT/PSS and reduced graphene oxide (RGO) was prepared using the freeze-drying technique (Figure 11a). The structural and morphological characterization results revealed that the PEDOT/PSS polymer formed a mesh-like structure, effectively covering the graphene layer, thereby providing support and enhancing the mechanical strength and electrochemical performance of the 3D material through a reduction in pore size (Figure 11b). The PEDOT–PSS/RGO composite sponge exhibits a significantly high electrical conductivity of 158 S·cm^−1^, making it suitable for use as an electrode. Additionally, its durability is enhanced by a remarkable factor of 700. The MB-ssDNA probes, labeled with single-stranded DNA (ssDNA) and methylene blue (MB), were immobilized onto a PEDOT–PSS/RGO composite sponge. Subsequently, electrochemical assays were conducted under optimized conditions (Figure 11c). The detection limit of the sensor can be reduced to 17 fM, and the linear range spans from 5 × 10^−11^ to 2 × 10^−3^ mM, rendering it suitable for the detection of hybrid DNA.

**Figure 12:**
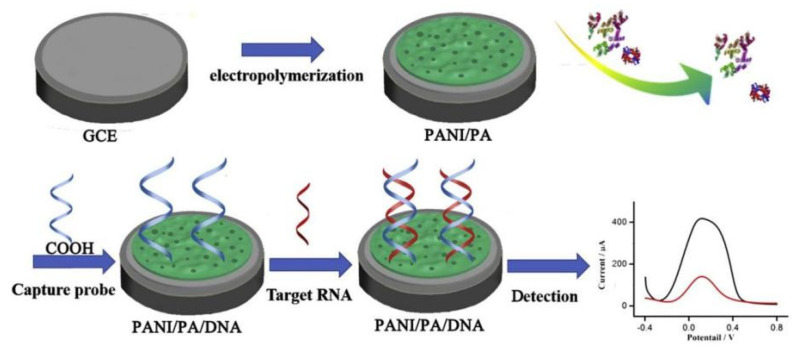
The structure of PANI/PA interface and its application for miRNA sensor. Reprinted with permission from ref. [105].

The PANI/PA hydrogels with a mesoporous structure were synthesized by Yang using a one-step electrochemical method and have been utilized for the detection of microRNA [105]. The biosensor was developed by immobilizing the DNA probe onto the PANI/PA interface and utilizing the REDOX current of PANI as the sensing signal for detecting DNA/RNA hybridization reaction (Figure 12). Due to the characteristic properties of PANI/PA hydrogels, this biosensor exhibits a wide linear range (1 × 10^−12^–1 × 10^−9^ mM), a low detection limit (0.34 fM), and efficient detection capability for microRNA mismatch. Importantly, PANI/PA hydrogels not only offer a plethora of active sites for DNA immobilization but also generate inherent electrochemical signals through the reduction and oxidation reactions in PANI, eliminating the need for additional REDOX probes.

#### 2.1.5. Other Substances

The range of biomolecules is extensive, encompassing tryptophan [106,107], dopamine [108,109], and various others in addition to those previously mentioned. Among the crucial indicators that facilitate various physiological processes, tryptophan plays a vital role in protein synthesis and serves as a significant precursor to serotonin. Being an indispensable amino acid, tryptophan exerts profound influence on human health [110,111]. Xu’s team incorporated PEDOT/PSS into a zionotropic PSBMA (poly-sulfobetaine methacrylate) network to fabricate an innovative semi-interpenetrating hydrogel [112]. This versatile hydrogel lays the foundation for the development of a stain-resistant wearable molecularly imprinted sensor capable of sensitive and robust detection of tryptophan in complex sweat. Dopamine modulates motor, affective, cognitive, and endocrine functions within the central nervous system, while also playing a role in the regulation of vasoconstriction. Rishabh Bansal’s team prepared the fabrication of a flexible sensor based on a hybrid nanocomposite of self-supporting polypyrrole electrode modified copper nanoparticles (PPy-Cu) for the electrochemical detection of dopamine [113]. The sensing capability of the self-standing PPy-Cu electrode was evaluated by chronoamperometry and optimized for different copper deposition times. PPy-Cu 120 has good dopamine detection performance. The lower limit of detection was 1.19 µM, and the linear range was 2.5 × 10^−3^–2.5 × 10^−1^ mM. In addition, this self-standing sensor is entirely composed of polypyrrole (a biocompatible polymer) and copper nanoparticles, making it sustainable and environmentally friendly. These encouraging results pave the way for the development of the next generation of flexible sensors that detect neurotransmitters and environment-related analytes.

### 2.2. Cancer Markers

The accurate detection of disease biomarkers in human body fluids, such as blood, urine, and saliva, with high sensitivity and specificity is crucial for early disease diagnosis and effective treatment [114,115,116]. The immunochemical methods based on antigen–antibody interactions have been widely adopted in daily clinical practice as the preferred approach for sensitive and reliable detection of protein-based cancer biomarkers in body fluids [117,118,119]. Over the years, various immunochemical methods have been developed, with electrochemical immune sensors being the preferred choice among scientists. However, the limited conductivity, small surface area, and poor anti-interference capabilities of general electrochemical immune sensors have hindered their development. The issue was addressed by scientists through the introduction of CPs, which fix antibodies within a network structure of these polymers using binders. This approach significantly increases the contact area and accelerates electron transmission while enhancing anti-interference ability [120,121].

Aydin developed an immunosensor utilizing gold nanoparticles/amino-functionalized thiophene polymer (AuNPs/P(ThiAmn)) for impedance-based detection of GM2 activating protein (GM2A) [122]. The AuNPs/P(ThiAmn) multilayer films were fabricated using electrochemical synthesis technology, thereby yielding conductive multilayer films suitable for biosensor applications. Due to the increased surface area and enhanced electron transfer properties of AuNPs and conjugated polymers, numerous anti-GM2A antibodies were attached to the amino group of P(ThiAmn) polymers. As a result, the modified electrode surface was prepared for the selective analysis of GM2A. Even in the presence of other interfering biomolecules, the proposed biosensor exhibits exceptional analytical detection performance with high sensitivity and specificity towards GM2A. Under optimal conditions, electrochemical impedance spectroscopy (EIS) enables determination of GM2A within a linear concentration range of 0.0185–111 pg·mL^−1^, achieving a limit of detection as low as 5.8 fg·mL^−1^. Similarly, Zhao et al., utilized AuNPs embedded within multiple 3D layers in various CPs substrates, such as poly (thiophen-3-acetic acid), poly (pyrrolio-2-carboxylic acid), and poly (pyrrolio-3-carboxylic acid), exploiting their extensive surface area and superior conductivity as transducers [123]. The identification of Amyloid-β oligomers (AβO), which are believed to be responsible for the neurotoxic effects associated with Alzheimer’s disease, was specifically accomplished by utilizing cellular prion protein (PrPC) as a biometric component. The PrPC/AuNPs embedded PPy-3-COOH matrix exhibits enhanced sensitivity and an extended detection range (10^−15^–10^−9^ mM). Additionally, a high-performance sensor based on a double-template molecularly imprinted polymer has been successfully utilized for the specific detection of lung cancer biomarkers carcinoembryonic antigen (CEA) and alpha-fetoprotein (AFP) [124]. The impedance method was employed to detect the rebinding of template antigens, wherein an increase in charge transfer resistance was observed with escalating concentrations of CEA and AFP. The linear dynamic ranges for CEA and AFP were 5–10^4^ and 10–10^4^ pg·mL^−1^, respectively, while the detection limits stood at 1.6 and 3.3 pg·mL^−1^.

**Figure 13:**
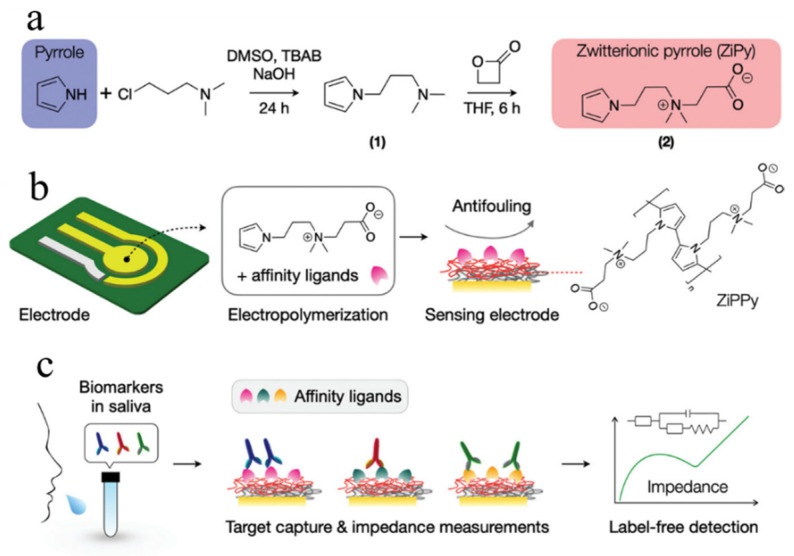
(**a**) Pyrrole is reacted with 3-dimethylaminopropylchloride hydrochloride to produce N, N-dimethyl-3-(1H-pyrrol-1-yl) propan-1-amine and then converted to ZiPy. (**b**) A mixture of affinity ligands and ZiPy monomers is drop-cast on electrodes. (**c**) Antibodies (target biomarker) present in saliva are captured by different antigens immobilized on ZiPPy-modified electrodes. Reprinted with permission from ref. [125].

Kilic has developed a novel CPs material called zwitterionic polypyrrole (ZiPPy), which offers optimal surface conditions for biosensing electrodes (Figure 13) [125]. ZiPPy possesses two distinct advantages: the zwitterion functionality effectively hydrates the electrode surface and impedes non-specific binding of hydrophobic proteins. Compared to bare and PPy-modified electrodes, the ZiPPy-modified electrode exhibits lower electrochemical impedance and reduced non-specific protein adsorption (low dirt). Furthermore, in a one-step electropolymerization process, affinity ligands for target biomarkers can be immobilized with ZiPPy. Specifically, a ZiPPy-modified electrode was designed for the detection of severe acute respiratory syndrome Coronavirus 2 (SARS-CoV-2), achieving a LOD as low as 5 × 10^−4^ pg·mL^−1^ in human saliva without the need for sample purification or secondary labeling.

### 2.3. Drug Screening and Delivery

The role of CPs-based sensors in drug screening and delivery is to monitor drug release in real time, assess drug activity and efficacy, and evaluate the biocompatibility of drug carrier materials to support precision therapy.

Bipolar disorder is a chronic psychiatric condition that imposes significant social and economic burdens, characterized by pronounced mood fluctuations in affected individuals [126,127]. Valproic acid is the first-line pharmacological treatment for stabilizing daily mood in patients with bipolar disorder [128]. However, elevated levels of valproic acid in the bloodstream can lead to severe adverse reactions, necessitating regular monitoring of blood valproic acid concentrations in patients [129]. Yuan et al., developed an innovative electrochemical sensor for the selective and facile detection of valproate, utilizing a molecularly imprinted polymer film prepared via one-step electro-polymerization [130]. The binding of the target molecule to the custom bionic PPy membrane of valproic acid obstructs the cavity within the membrane and induces alterations in its electrical properties, which can be detected through differential pulse voltammetry (DPV) as a reduction in peak current. The peak current changes exhibited a strong logarithmic response to the concentration of valproate. Similarly, tramadol (TRA) is a weak opioid analgesic utilized for the management of mild to moderately severe pain [131]. However, excessive use of TRA has been frequently associated with adverse effects such as vomiting, depression, tachycardia, convulsions, morbidity, and mortality [132]. Diouf developed an electrochemical sensor based on a molecularly imprinted conductive polymers (MICP) for the quantitative and non-invasive detection of TRA [133]. The MICP-based sensor was fabricated by employing self-assembly techniques to deposit a layer of PANI coated with silver nanoparticles (AgNPs) onto a screen-printed gold electrode (Au-SPE), followed by polymerization of 2-aminothiophene in the presence of TRA. Under optimized conditions, the response of this sensor is directly proportional to the concentration of TRA within the range from 1 × 10^4^ to 1 × 10^8^ pg·mL^−1^.

An updated comparative list of different CPs applications in biomedicine is presented in Table 1.

## 3. Environmental Applications

Electrochemical sensors based on CPs have been extensively utilized in environmental monitoring, encompassing the determination of nitrate nitrogen, heavy metals, antibiotics, and pesticides in soil and water environments. Additionally, they have also been employed for the detection of toxic gases.

### 3.1. Nitrate Nitrogen

The expansion of industrialization and excessive fertilizer use in agriculture contribute to the escalation of nitrate pollution in water and soil [106,134]. Overuse of nitrate leads to increased algae growth, contamination of groundwater and surface water, and ecological damage. Moreover, excessive intake of nitrate by humans can cause various complications [135]. Bacterial enzymes convert nitrate to nitrite in the human digestive system, disrupting hemoglobin’s oxygen transportation function and causing symptoms of hypoxia [136,137]. Additionally, it reacts with amines in the gastrointestinal tract to produce carcinogens [138]. Nitrate presence can also lead to other health issues such as headaches, nausea, and vomiting. Therefore, developing an accurate and efficient method for detecting nitrate is crucial.

Both Zhang’s and Motaghedifard’s teams have developed all-solid ion selective electrode (ASS-ISE) that incorporate CPs and other nanoparticles for the detection of nitrate ion [139,140]. While the main difference lies in the selection of modified CPs at the electrode interface. The sensor developed by Zhang’s group utilizes a molecularly imprinted PPy membrane and exhibits a linear range of 5.25 × 10^−2^–1 × 10^2^ mM, with a response slope of −50.4 mV·dec^−1^ [139]. The molecularly imprinted PPy membrane significantly improves the limitations of the PVC sensitive membrane, while incorporating AuNPs to enhance electrical conductivity and promote stronger bonding properties. Motaghedifard’s research group developed a hybrid nanocomposite of Au-modified MWCNT/Cu/PANI for the detection of nitrate ions [141]. This sensor utilizes precious metal bimetallic nanoparticles (gold and copper) to catalyze the reduction of nitrates in polymer carbon modifiers, achieving a remarkable detection limit of 0.09 µM. The electrode has been successfully employed for the determination of nitrate ions in both industrial wastewater and aqueduct water from a barium chromate production line.

The continuous measurement of nitrate nitrogen holds significant potential applications in the fields of plant biology, plant breeding, environmental science, and agricultural production. In contrast to the aforementioned preparation of ASS-ISEs, Ali reported a novel ASS-ISE for real-time continuous monitoring of soil nitrate levels [142]. The patterned gold WE of this sensor was modified with poly(3-octylthiophene) and molybdenum disulfide (POT-MoS2). The POT-MoS2 layer acts as an ion-to-electron transduction layer, possessing high hydrophobicity and REDOX properties. This layer significantly enhances conductivity and anion exchange while minimizing the formation of a thin water layer at the interface between the Au electrode and the ion-selective membrane (ISM). Consequently, this sensor exhibits exceptional performance in terms of selectivity, sensitivity, and long-term stability even when exposed to significant concentrations of other anions. Vapor phase polymerization (VPP) is a common technique for directly polymerizing gaseous monomers into polymers under gas phase conditions [143]. In the VPP process, monomer gases undergo a series of chemical reactions in the gas phase to form a polymer film or coating [143]. By employing VPP technology, Kohler’s group prepared a real-time sensor based on PEDOT for the detection of nitrate ions through monitoring changes in electrical properties [144]. The sensitivity of the sensor to nitrates is influenced by various polymerization parameters such as temperature, pressure, and time. Under optimized conditions, the sensor exhibits a resistance response of 41.79% to a nitrate solution concentration of 1000 ppm. Furthermore, it has the capability to detect nitrate concentrations ranging from 1 ppm to 1000 ppm. The proposed sensor demonstrates significant potential for the real-time monitoring of excessive nitrate ions in aqueous solutions.

### 3.2. Heavy Metal Ions

The presence of heavy metal ions (HMIs) in water, soil, and other natural environments is a significant environmental concern. These pollutants originate from various sources such as nature, industrial discharges, agricultural activities, sewage treatment plants, and drinking water pipes. Due to their high toxicity and non-degradability, HMIs pose serious threats to the ecological environment as well as public and human health. The toxic metal ions, such as cadmium (Cd^2+^), lead (Pb^2+^), mercury (Hg^2+^), cobalt (Co^2+^) and others, exhibit high mobility in aquatic environments and have a propensity for bioaccumulation along the food chain, thereby posing a potential risk to human health even at low concentrations [10,145]. The detrimental effects of HMIs on the human body are mediated through various mechanisms, including induction of oxidative stress, DNA damage, impairment of protein function, initiation of cell apoptosis, and promotion of inflammatory reactions [146].

**Figure 14:**
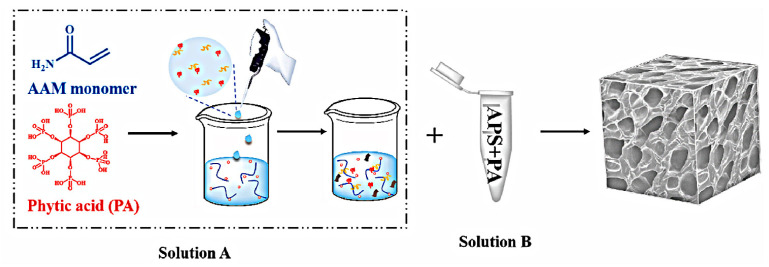
Schematic diagram of g-C_3_N_4_-P(ANI-Py)-PAAM polymer hydrogel synthesis. Reprinted with permission from ref. [147].

Xiao et al., designed and prepared a 3D high-porosity CPHs-based sensor, which successfully enabled the detection of Cd^2+^, Cu^2+^, Pb^2+^ and Hg^2+^ [147]. The synthesis of a g-C_3_N_4_-P(ANI-Py)-PAAM polymer hydrogel involved the crosslinking of aniline pyrrole copolymer with acrylamide, phytic acid as both dopant and crosslinking agent, followed by integration with g-C_3_N_4_ (Figure 14). The 3D mesh high-porosity hydrogels not only exhibit excellent electrical conductivity, but also offer a substantial surface area for augmenting the population of immobilized ions [148]. The LOD for Cd^2+^, Cu^2+^, Pb^2+^ and Hg^2+^ by this sensor were determined to be 2.608 × 10^−5^ mM, 5.323 × 10^−5^ mM, 1.484 × 10^−5^ mM and 6.835 × 10^−5^ mM, respectively, making it suitable for trace analysis. In addition, the sensor demonstrates exceptional accuracy in lake water quality assessments. The preparation and implementation of hydrogels in electrochemical sensors offer a viable approach for the electrochemical capture and detection of diverse hazardous materials in solution, thereby presenting significant prospects for commercial applications. The group led by Kulkarni has also developed a sensor utilizing PPy to detect HMIs, effectively combining the exceptional properties of graphene nanoribbons (GNR) with conductive PPy [149]. GNR was synthesized via the MWCNT decompression method, while GNR/PPy was synthesized through in situ polymerization. The material comprises multiple layers of carbon and nitrogen that facilitate binding to Pb^2+^. The modified electrochemical sensor exhibited significantly enhanced peak current intensity for the rapid detection of Pb^2+^, achieving a remarkable detection limit of 0.03 nM. Moreover, the electrode demonstrated exceptional selectivity towards Pb^2+^, even in the presence of Cu^2+^, Zn^2+^, and Hg^2+^.

The utilization of PANI as an interface modification material for HMIs detection is also investigated [150]. The composites of polyaniline-benzothiazole [(3,5-bis(benzo[d]thiazol-2-yl)-[1,1–biphenyl]-4-ol)] (PANI-BEN) were synthesized for the detection of Hg^2+^ and Pb^2+^, achieving detection limits of 1pM and 4.6pM, respectively [151]. Some researchers have investigated the combination of metal–organic frameworks (MOFs) and PANI [152]. PANI was synthesized in both conductive (emerald salt, ES) and non-conductive forms (emerald base, EB), with six different mass ratios of MOF-5 and PANI used to produce the conjugated polymer. The electrochemical response of all composites towards the presence of Cd^2+^ and Pb^2+^ is consistently observed. Among them, the MOF/EB-1 composite with 71.0 wt.% MOF-5 exhibits the highest oxidation current when both HMIs are detected individually or simultaneously. The current density recorded by MOF/EB-1 is also higher than that of its individual components, indicating a synergistic effect wherein MOF-5 offers a substantial surface area for the adsorption of Cd^2+^ and Pb^2+^, while PANI facilitates an electron transfer network during the subsequent metal oxidation.

**Figure 15:**
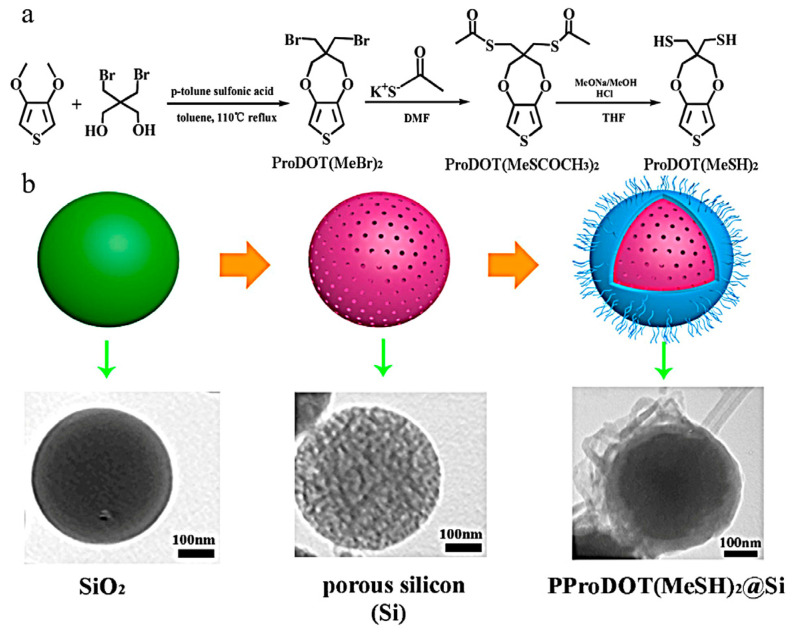
(**a**) The synthesis route of double-thiol linked (3,4-proplenedioxythiophene) ProDOT(MeSH)_2_ monomer. (**b**) PProDOT(MeSH)_2_@Si synthesis route. Reprinted with permission from ref. [153].

Furthermore, a novel composite consisting of a mercaptan (-SH) grafted polymer (3,4-propenedioxthiophene)/porous silicon sphere (PProDOT(MeSH)_2_@Si) has been employed for the electrochemical detection of Cd^2+^, Pb^2+^ and Hg^2+^ (Figure 15) [153]. The PProDOT(MeSH)_2_@Si composite was synthesized via chemical oxidation polymerization, and the detection of Cd^2+^, Pb^2+^ and Hg^2+^ was performed using differential pulse voltammetry (DPV). The results demonstrated that the incorporation of two sulfhydryl chains into the monomer unit enhances the electrochemical sensitivity of PProDOT(MeSH)_2_, which can be attributed to the interaction between sulfhydryl (-SH) groups and heavy metal ions. Moreover, the integration of PProDOT(MeSH)_2_ with porous Si sphere significantly improves the electrocatalytic efficiency of the electrode material.

### 3.3. Antibiotics

Concern over the hazardous accumulation of antibiotics in food and water resources has significantly increased in the past decade. The excessive use and improper disposal of antibiotics, as well as the discharge of animal and human waste, aquaculture practices, hospital and industrial waste, along with inefficient removal during conventional water treatment processes contribute to the build-up of antibiotics in both food and the environment. Consequently, antibiotics have been identified as an emerging environmental pollutant in recent years [154,155,156].

Cefixime (CEF, a semi-synthetic antibiotic, third-generation cephalosporin) exhibits bactericidal properties by inhibiting bacterial cell wall formation, growth, and proliferation, ultimately leading to bacterial death [157]. A novel electrochemical sensor was developed by incorporating expanded graphene oxide and gold nanowires, followed by the electrical polymerization of PANI MIPs on the electrode surface [158]. This sensor demonstrates linear response within a concentration range of 2 × 10^−5^–9.5 × 10^−4^ mM with a detection limit of 7.1 nM.

Sulfonamides (SAs) are a class of broad-spectrum synthetic antibacterial and anti-inflammatory antibiotics, including sulfamethizole, sulfadiazine, and sulfisoxazole. Due to their high efficacy and cost-effectiveness, SAs have been extensively utilized for the prevention and control of human and animal infections as well as for promoting animal husbandry growth [159]. Kong’s group demonstrated a reusable and label-free electrochemical sensor utilizing an electropolymeric MIP film as an identification layer for rapid and sensitive detection of sulfamethozole [155]. In order to achieve effective identification, computational simulation and subsequent experimental evaluation were conducted for monomer screening of four 3-substituted thiophenes, ultimately selecting 3-thiophene ethanol. The synthesis of MIPs is rapid and environmentally friendly, allowing for in situ manufacturing on the sensor surface. Under optimized experimental conditions, the sensor exhibits a good linear relationship within the range of 1 × 10^−6^–1 × 10^−2^ mM, with a detection limit as low as 0.18 nM.

Oxacillin (OXC) is a penicillin-based antibiotic commonly employed for the treatment of infections caused by susceptible strains, including those affecting the respiratory tract, skin, soft tissues, and other infection symptoms. The mechanism of action of oxacillin involves inhibiting bacterial cell wall synthesis, ultimately leading to bacterial death [160]. The screen-printed carbon electrode (SPCE) was utilized to fabricate an oxacillin sensor. The SPCE underwent modification with nano-gold and graphene oxide, followed by electropolymerization of aniline in the presence of graphene oxide [156]. Under a typical peak potential of 0.82 mV (relative to Ag/AgCl), the response exhibited a linear range spanning from 7 × 10^−7^ to 5.75 × 10^−4^ mM. The sensitivity was determined as 9.76 × 10^4^ μA·mM^−1^·cm^−2^, while the detection limit stood at 2 × 10^−7^ mM. In the case of a concentration of OXC at 2 × 10^−4^ M, the relative repeatability for six repeated experiments was found to be 2.6%.

Dimetronidazole (DMZ), a derivative of nitroimidazole, is a veterinary drug used as an antibiotic to treat bacterial or protozoan infections in poultry. The electrochemical detection of DMZ was conducted using SPCE modified with PANI-Cu@BSA/RGO nanocomposites [161]. The PANI-Cu@BSA electrocatalyst was synthesized through one-step biomimetic mineralization polymerization, utilizing bovine serum albumin (BSA) as a stabilizer. And the PANI-Cu@BSA/reduced graphene oxide (RGO) nanocomposites were prepared through ultrasonic coating, exhibiting exceptional water dispersibility, high electrical conductivity, and nano-sized particles. The increase in current intensity primarily stems from the enhanced rapid electron transfer between the electrode and the analyte, facilitated by the combination of PANI-Cu@BSA with RGO. This synergistic effect leads to an exceptional electrical conductivity and active surface area at the analyte–electrode junction.

### 3.4. Pesticide

Pesticide residues can pose various risks to human health, primarily through the long-term consumption of food containing such residues, which may result in chronic poisoning and adversely affect the nervous system, endocrine system, immune system, among others. This can lead to chronic diseases like cancer and nervous system dysfunction. Children and pregnant women are particularly vulnerable to pesticide residues as their growth and development can be negatively impacted by prolonged exposure, increasing the risk of adverse effects and disease. Furthermore, pesticide residues not only harm human health but also contribute to soil and water pollution while disrupting ecological balance [162].

To tackle this problem, researchers have devised electrochemical sensors utilizing CPs to detect pesticide residues. The electrochemical sensor developed by Anirudhan utilized a combination of MWCNT and CPs to effectively detect chlorpyrifos (CPF) in vegetable sample solutions [163]. The copolymerization of 3-thiophenic acid and 3,4-ethylenedioxythiophene was conducted on the surface of MWCNTs, followed by drop-casting onto the electrode surface for highly sensitive detection of CPF. Under optimal conditions, the sensor has an extremely low detection limit of 0.004 nM. The detection of glyphosate (Gly) was accomplished by Ding through the utilization of molecularly imprinted PPy nanotubes, which were fabricated by imprinting Gly binding sites onto the surface of PPy nanotubes [164]. The modified SPCE electrode exhibited excellent conductivity and rapid adsorption rate, as well as enhanced affinity and specificity towards Gly. The portability of this sensor makes it highly suitable for real-time detection, as evidenced by its successful application in detecting Gly in orange juice and rice beverage samples. This demonstrates its efficacy for pesticide monitoring in practical food and environmental matrices. Similarly, Deller proposes a novel electrochemical sensor based on modified SPCE to quantify Pirimicarb (PMC), a carbamate pesticide that is challenging to detect electrochemically due to its high oxidation potential [165]. To enhance the electrochemical performance for PMC oxidation at low potential, SPCE was modified with conductive PEDOT and AuNPs. This modification enables the exploration of the correlation between oxidation peak and pesticide concentration through an electrocatalytic effect, leading to the development of a sensitive, selective, and cost-effective electrochemical sensor for future environmental monitoring.

### 3.5. Toxic Gases

Humans are exposed to a wide range of air pollutants in both indoor and outdoor environments. Poor air quality is recognized as a trigger for various health issues that often result in life-threatening and costly emergency medical care [166]. Therefore, accurate detection of toxic gases would not only bring significant benefits to industries but also greatly improve the daily lives of individuals.

Ammonia is harmful to the respiratory system, skin and eyes, digestive system and nervous system, and also causes environmental pollution [167]. The PPy-based gas sensor for NH_3_ detection, developed by Jain, utilizes a combination of oxidizer and pyrrole to achieve exceptional thermal stability, superior electrical conductivity, and environmental stability [168]. The chemical oxidation polymerization method was employed to synthesize PPy in an aqueous solution, utilizing ferric chloride (FeCl_3_) as the oxidant and cetrimonium bromide as the solvent [169]. With the increase in the molar ratio of FeCl_3_ to pyrrole, the conductivity of PPy increased, and the conductivity of PPy was analyzed by I-V characteristics to get the optimal ratio. The influence of different concentrations of NH_3_ on the conductivity of PPy was used to achieve the detection of NH_3_. While Bhardwaj prepared a three-phase Cu-MOF/graphene/PANI composite for NH_3_ sensing [170]. The three-phase composite has been synthesized in the presence of ammonium persulfate as an oxidizing agent. In this mixture, the aniline component undergoes polymerization to form PANI, which acts as a connecting bridge between Cu-MOF and graphene within the composite. The incorporation of Cu-MOF into the matrix facilitates the attainment of an electrochemically active sensing material possessing a surface area of approximately 756 m^2^·g^−1^, which is advantageous for NH_3_ diffusion. Moreover, this porous PANI-based sensor demonstrates remarkable sensitivity towards NH_3_ detection, exhibiting linear detection ranges spanning from 1 to 100 ppm and low detection limits of 0.6 ppm. In addition to PPy and PANI, PEDOT has also been utilized for the detection of NH_3_. A high-performance chemically resistant NH_3_ sensor was developed by preparing an ultra-thin Janus film consisting of AuNWs/PEDOT–PSS double layers [171]. The AuNWs/PEDOT–PSS Janus film was fabricated by subjecting the AuNWs membrane to H_2_S treatment, resulting in a hydrophilic surface, which was subsequently combined with a layer of PEDOT/PSS. The Janus film containing a single layer of AuNWs exhibited the most pronounced response to NH_3_. Furthermore, as the thermal annealing treatment temperature increased within the range of 80 to 200 °C, the sensitivity towards NH_3_ also escalated due to the alteration in the state of AuNWs, leading to an enlargement in available surface area for NH_3_ adsorption. Significantly, this study introduces a novel approach for fabricating flexible, transparent, and conductive Janus films, thereby establishing a promising platform for electrochemical sensors.

H_2_S is a gaseous compound of hydrogen and sulfur, characterized by a potent odor and exhibiting toxic effects on the respiratory system, nervous system, and biomolecules in humans [172]. The biodegradable electroactive polymer, polyurethane-urea (PUU) and PUU-activated carbon (AC) composites were utilized as sensitive materials for successfully fabricate a high-performance H_2_S sensor capable of operating at room temperature [173]. The PUU was synthesized through the copolymerization of biodegradable polycaprolactone diol and electroactive amine-coated aniline trimers, while the activated carbon was prepared from waste coconut shell. The investigation of PUU and AC was conducted in varying proportions, resulting in a higher sensitivity to H_2_S gas when AC was added. It is hypothesized that the enhanced response of the PUU-AC composite to H_2_S can be attributed to its elevated surface area and abundant reaction sites, which facilitate gas diffusion, adsorption, and electron transfer. The successful fabrication of interdigitated electrode (IDE) coated with PANI-AC composites enables the homoplastic detection of H_2_S [174]. The PANI was synthesized through oxidative coupling polymerization of aniline monomers, while the AC was obtained by calcinating coconut husks from agricultural waste at 800 °C with ZnCl_2_ as a catalyst. The superior gas-sensitive properties of PANI-AC composites can be attributed to the increased surface area, providing more active sites for doping and enhancing sensing capability. Specifically, incorporating AC into the PANI matrix significantly improves the doping process, resulting in a stronger response to H_2_S, higher repeatability, and greater stability compared to pure PANI-coated IDE sensors.

**Figure 16:**
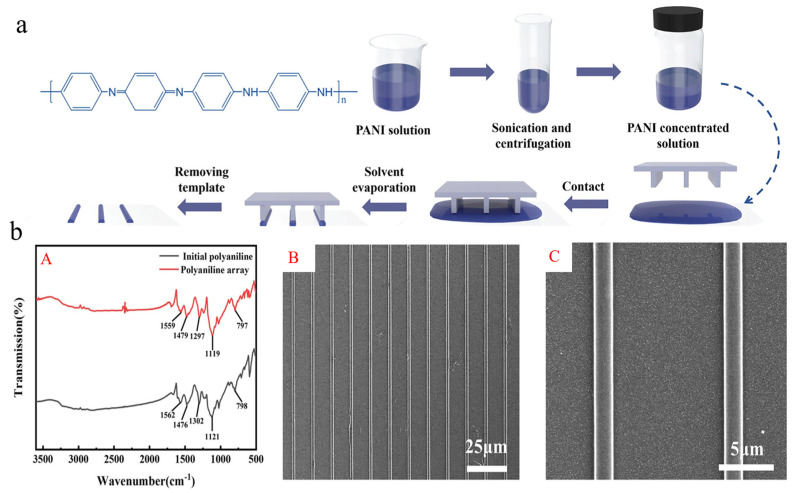
(**a**) Schematic diagram of PANI microwire array prepared by liquid film-induced capillary bridge method. (**b**) FTIR spectra of the initial PANI (black) and PANI array (red) (**A**). The FTIR spectra of the two samples were consistent, indicating that the PANI micron array was prepared by liquid film-induced capillary bridge method. SEM image of PANI array with microarrays arranged in parallel and evenly spaced (**B**). SEM image of PANI array after partial amplification (**C**). The edge of the microwire is straight and the width is uniform. Reprinted with permission from ref. [178].

PANI is a potential material for electrochemical sensors because of its special physical and chemical characteristics, which include outstanding gas absorption, little dielectric loss, and exceptional durability in both chemical and thermal conditions. However, the sensing performance is highly dependent on the structure and size of PANI [175,176,177]. Although in situ oxidation polymerization combined with self-assembly processes has become the primary method for fabricating flexible PANI-based gas sensors, achieving a uniform array of microwires remains an urgent challenge that needs to be addressed. Zhao adopted a novel approach to fabricate one-dimensional PANI microwire arrays via the liquid film-induced capillary bridge method, utilizing an extremely low concentration of PANI solution [178]. The formation rate of PANI microwire can be significantly enhanced by employing a silicone template modified with hydrophobic 1H,1H,2H,2H perfluorodecyl triethoxysilane (PTES) (Figure 16). The PANI solution, with a concentration of 1 × 10^12^ pg·mL^−1^ and a silicon template of 10 μm, was evaporated at 80 °C for 18 h, resulting in a 100% rate of PANI microwire formation. The obtained PANI microwires demonstrate uniformity in size and possess well-defined shapes, with respective widths and heights measuring 2 μm and 250 nm (Figure 16b). The prepared microwire array enables the detection of SO_2_ at room temperature, exhibiting a response time of 20 s and a detection limit of 1 ppm. As a result, the liquid film-induced capillary bridge method offers an innovative opportunity for fabricating gas sensor devices based on insoluble polymers.

**Figure 17:**
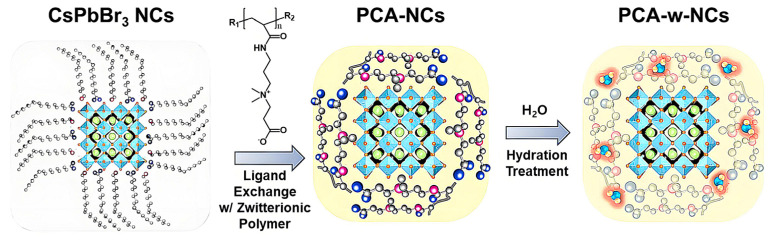
Schematic diagram of CsPbBr_3_ perovskite NCs being exchanged by polymer ligands. Reprinted with permission from ref. [179].

Jang has created a highly sensitive OFET-based gas sensor to detect NO_2_. The conducting polymer poly (3-hexylthiophene)(P3HT) acts as a charge transport agent and is placed in a semiconductor layer with a field effect transistor (OFET) [179]. The surface chemistry of the perovskite NCs is modified by ligand exchange treatment so that the NCs is encased in a photoionic polymer that strongly interacts not only with the surface of the perovskite NCs, but also with the target gas molecules (Figure 17). In addition, the researchers used hydrated perovskite NCs to optimize their ability to capture NO_2_ through interactions with water molecules and NO_2_. These results demonstrate the potential of organic–inorganic hybrid sensors, which will provide a new approach for developing gas sensors based on composites of different materials, aimed at increasing sensitivity and selectivity.

An updated comparative list of different CPs applications in environmental monitoring is presented in Table 2.

## 4. Summary and Prospect

Recent research has significantly advanced our understanding of the potential of CPs in electrochemical sensors, which holds great promise for revolutionizing current practices in medical care and environmental monitoring by shifting the focus from traditional off-site laboratory analysis to in situ detection. CPs can be functionalized with diverse groups, enabling their transformation into a wide range of biometric molecules. Furthermore, integration with organic/inorganic nanomaterials enhances their sensitivity and selectivity in detecting biological analytes.

Although CPs-based electrochemical sensors have advantages in biomedical and environmental applications, there are still areas for improvement, such as CPs being affected by oxidation, hydrolysis, light, and other factors, resulting in limited stability and service life. Some CPs may be too sensitive to specific molecules under certain conditions, causing the presence of interfering substances to interfere with the accuracy of the sensor. Some CPs are difficult to regenerate or recycle, resulting in resource waste and environmental burden. Therefore, improving the reliability and applicability of electrochemical sensors is an important research field, which involves many aspects of optimization. In terms of improving sensitivity, the signal response of the sensor can be enhanced by optimizing the electrode design and material selection of the sensor. Increasing specificity requires selectively improving the properties of the target analyte, such as surface modifications or the introduction of molecular recognition elements. Long-term stability can be achieved by preventing contamination and oxidation of the electrode surface. In terms of biocompatibility, materials compatible with the organism should be selected to ensure the safe and reliable application of the sensor in the organism. In terms of practicality and portability, miniaturization technology and portable test platforms can be used to simplify the operation process and improve the ease of use. Finally, in terms of cost-effectiveness, costs can be reduced by optimizing material selection, the manufacturing process and production scale, thus promoting the commercialization and scale application of electrochemical sensors. It is believed that with the development of the CPs field, new conductive polymers can be designed to improve their stability, selectivity and repeatability to meet the needs of different application fields. Intelligent technologies such as artificial intelligence and machine learning can be combined to optimize sensor response and performance and improve accuracy and reliability. The development of multifunctional CPs capable of simultaneously detecting multiple target substances has improved the applicability and practicality of sensors. The preparation method of environmentally friendly CPs was studied to reduce energy consumption and environmental pollution and promote the sustainable development of electrochemical sensors. In the future, CPs have broad application prospects in the field of electrochemical sensors, but the field also needs continuous technological innovation and improvement to solve existing defects and meet the needs of future applications.

## Figures and Tables

**Table 1:** The main electrochemical sensors based on CPs and their parameter analysis, the application of electrochemical technology and its application in biomedical detection are introduced.

CPs	Analyte	LOD (mM)	Linear Range(mM)	Application	Ref.
PANI	PH	0.0014	10^−5^–0.1	Biomolecular detection	[70]
PANI	pH	0.0016	8 × 10^−6^–0.1	Biomolecular detection	[74]
PANI	Glucose	0.0007	0.01–8	Biomolecular detection	[86]
PANI	Glucose	0.2	1–25	Biomolecular detection	[87]
PEDOT/PSS	Glucose	0.0019	10^−3^–0.094	Biomolecular detection	[88]
PEDOT/PSS	Glucose	0.0009	0.094–1.294	Biomolecular detection	[89]
PPy	Cholesterol	0.1	0.0002–0.5	Biomolecular detection	[95]
PANI	Cholesterol	0.00095	2–8	Biomolecular detection	[96]
PEDOT/PSS	DNA	1.7 × 10^−11^	0.003–1	Biomolecular detection	[104]
PANI	microRNA	3.4 × 10^−13^	5 × 10^−11^–2 × 10^−3^	Biomolecular detection	[105]
PEDOT/PSS	tryptophan	0.00067	1 × 10^−12^–1 × 10^−9^	Biomolecular detection	[112]
PPy	Dopamine	0.00119	0.002–0.1	Biomolecular detection	[113]
PTH	GM2A	2.13 × 10^−11^	2.5 × 10^−3^–2.5 × 10^−1^	Cancer makers	[122]
PPy	AβO	10–15	6.79 × 10^−11^–4.07 × 10^−7^	Cancer makers	[123]
PPy	CEA, AFP	5.87 × 10^−12^, 1.21 × 10^−11^	10^−15^–10^−3^	Cancer makers	[124]
PPy	SARS-CoV-2	1.84 × 10^−4^	1.83 × 10^−11^–3.67 × 10^−8^, 3.67 × 10^−11^–3.67 × 10^−8^	Cancer makers	[125]
PPy	Valprate	0.01748	5.5 × 10^−12^–3.67 × 10^−7^	Drug screening and delivery	[130]
PANI	TRA	0.0346	1.84 × 10^−14^–2.75 × 10^−13^	Drug screening and delivery	[133]

**Table 2:** The main electrochemical sensors based on CPs and their parameter analysis, the application of electrochemical technology and its application in environmental detection are introduced.

CPs	Analyte	LOD (mM)	Linear Range (mM)	Application	Ref.
PPy	nitrate	0.0537	0.0525–100	Nitrate nitrogen	[139]
PANI	nitrate	9 × 10^−5^	0.0008–0.03	Nitrate nitrogen	[141]
POT	nitrate	2.1 × 10^−5^	1.61 × 10^−5^–24.19	Nitrate nitrogen	[142]
PEDOT	nitrate	1.61 × 10^−5^	1.61 × 10^−5^–16.13	Nitrate nitrogen	[144]
PANI, PPy	Cd^2+^, Cu^2+^, Pb^2+^, Hg^2^	2.608 × 10^−5^, 5.323 × 10^−5^, 1.484 × 10^−5^, 0.835 × 10^−5^	0.0005–0.1, 0.001–0.1, 0.0001–0.1, 0.001–0.01	Heavy metal ions	[147]
PPy	Pb^2+^	3 × 10^−8^	10^−7^–10^−3^	Heavy metal ions	[149]
PANI	Pb^2+^, Hg^2^	4.6 × 10^−9^, 10^−9^	0.001–0.0248	Heavy metal ions	[151]
PANI	Cd^2^, Pb^2+^	6.85 × 10^−4^, 1.59 × 10^−4^	4.8 × 10^−6^–1.02 × 10^−5^, 1.11 × 10^−3^–1.93 × 10^−3^	Heavy metal ions	[152]
PEDOT	Cd^2+^, Cu^2+^, Pb^2+^	5.78 × 10^−6^, 2.7 × 10^−6^, 1.7 × 10^−6^	4 × 10^−5^–2.8 × 10^−3^, 2.4 × 10^−5^–2.8 × 10^−3^, 1.6 × 10^−4^–3.2 × 10^−3^	Heavy metal ions	[153]
PANI	CEF	7.1	2 × 10^−5^–9.5 × 10^−5^	Antibiotics	[158]
PTh	SAs	1.7 × 10^−7^	1 × 10^−6^–1 × 10^−2^	Antibiotics	[155]
PANI	OXC	2 × 10^−7^	7 × 10^−7^–5.75 × 10^−4^	Antibiotics	[156]
PANI	DMZ	1.78 × 10^−6^	7.9 × 10^−4^–2.057	Antibiotics	[161]
PTh	CPF	0.04 nM	2 × 10^−8^–10^−3^	Pesticide	[163]
PPy	GIy	7.12 × 10^−6^	9.18 × 10^−6^–1.28 × 10^−3^	Pesticide	[164]
PEDOT/PSS	PMC	0.02834	0.09381–0.75	Pesticide	[165]
PANI	NH_3_	3.52 × 10^−5^	5.87 × 10^−5^–5.87 × 10^−3^	Toxic gas	[170]
PEDOT/PSS	NH_3_	2.94 × 10^−5^	5.87 × 10^−4^–5.87 × 10^−3^	Toxic gas	[171]
PANI	H_2_S	5.87 × 10^−4^	2.93 × 10^−5^–1.47 × 10^−4^	Toxic gas	[173]
PANI	SO_2_	1.56 × 10^−5^	1.56 × 10^−5^–7.8 × 10^−4^	Toxic gas	[178]
P3HT	NO_2_	6.91 × 10^−12^	2.17 × 10^−4^–2.17 × 10^−2^	Toxic gas	[179]

## Data Availability

Not applicable.
